# The EIT-based global inhomogeneity index is highly correlated with regional lung opening in patients with acute respiratory distress syndrome

**DOI:** 10.1186/1756-0500-7-82

**Published:** 2014-02-06

**Authors:** Zhanqi Zhao, Sven Pulletz, Inéz Frerichs, Ullrich Müller-Lisse, Knut Möller

**Affiliations:** 1 Institute of Technical Medicine, Furtwangen University, Jakob-Kienzle Straße 17, D-78054 VS-Schwenningen, Germany; 2 Department of Anaesthesiology and Intensive Care Medicine, Medical Center Osnabrück, Osnabrück, Germany; 3 Department of Anaesthesiology and Intensive Care Medicine, University Medical Center Schleswig-Holstein, Kiel, Germany; 4 Department of Radiology, University of Munich, Munich, Germany

**Keywords:** Electrical impedance tomography, Acute lung injury, Global inhomogeneity index, Regional lung opening, Lung mechanics

## Abstract

**Background:**

The electrical impedance tomography (EIT)-based global inhomogeneity (GI) index was introduced to quantify tidal volume distribution within the lung. Up to now, the GI index was evaluated for plausibility but the analysis of how it is influenced by various physiological factors is still missing. The aim of our study was to evaluate the influence of proportion of open lung regions measured by EIT on the GI index.

**Methods:**

A constant low-flow inflation maneuver was performed in 18 acute respiratory distress syndrome (ARDS) patients (58 ± 14 years, mean age ± SD) and 8 lung-healthy patients (41 ± 12 years) under controlled mechanical ventilation. EIT raw data were acquired at 25 scans/s and reconstructed offline. Recruited lung regions were identified as those image pixels of the lung regions within the EIT scans where local impedance amplitudes exceeded 10% of the maximum amplitude during the maneuver. A series of GI indices was calculated during mechanical lung inflation, based on the differential images obtained between different time points. Respiratory system elastance (E_rs_) values were calculated at 10 lung volume levels during low-flow maneuver.

**Results:**

The GI index decreased during low-flow inflation, while the percentage of open lung regions increased. The values correlated highly in both ARDS (r^2^ = 0.88 ± 0.08, p < 0.01) and lung-healthy patients (r^2^ = 0.92 ± 0.05, p < 0.01). E_rs_ and GI index were also significantly correlated in 16 out of 18 ARDS (r^2^ = 0.84 ± 0.13, p < 0.01) and in 6 out of 8 lung-healthy patients (r^2^ = 0.84 ± 0.07, p < 0.01). Significant differences were found in GI values between two groups (0.52 ± 0.21 for ARDS and 0.41 ± 0.04 for lung-healthy patients, *p <* 0.05) as well in E_rs_ values (0.017 ± 0.008 cmH_2_O/ml for ARDS and 0.009 ± 0.001 cmH_2_O/ml for lung-healthy patients, *p <* 0.01).

**Conclusions:**

We conclude that the GI index is a reliable measure of ventilation heterogeneity highly correlated with lung recruitability measured with EIT. The GI index may prove to be a useful EIT-based index to guide ventilation therapy.

## Background

Ventilation distribution in patients under mechanical ventilation is often inhomogeneous [1]. Cyclic opening and closing of alveoli in patients with acute respiratory distress syndrome (ARDS) increases ventilation heterogeneity and the risk for ventilator-induced lung injury (VILI) [2]. Regional inhomogeneities of the damaged lung should be taken into consideration to develop improved ventilation strategies [3]. Unfortunately, hardly any established clinical tools possess the ability to assess regional ventilation in patients with ARDS except computed tomography (CT). However, CT is not suitable for bedside monitoring of patients with ARDS due to the radiation exposure and hazardous patient transport to the radiological examination site.

Electrical impedance tomography (EIT) is a non-invasive, radiation-free imaging technique. It measures regional lung ventilation and aeration distribution by means of changes in electrical potentials at the skin surface of the chest wall during breathing cycles [4]. The physical principle is that changes in lung ventilation and blood content in the thorax modify the electrical impedance of lung tissue [5,6]. Although EIT has a relatively low spatial resolution (typically an image with 32 × 32 pixels for EIT vs. 512 × 512 pixels for CT), it achieves a much higher temporal resolution than CT (typically 20–50 Hz for EIT vs. 0.3-1 Hz for CT) which is an important prerequisite for bedside monitoring. The reliability of EIT has already been confirmed by comparison with different established methods [7-9]. Applications of EIT have been reported in subjects with [10-13] and without [14-16] lung disease. However, EIT is still not widely accepted in the clinical environment due to the difficult interpretation of the results [17-20]. Understandable and clinically relevant analysis methods and EIT parameters are still warrant to improve the acceptance of EIT. There exist multiple approaches for defining such parameters: Wrigge *et al.,* Hinz *et al.* and Frerichs *et al.* considered the delay and the curvature of regional impedance-time curve as representing local lung mechanics [15,21,22]; Lowhagen *et al.* and Dargaville *et al*. analyzed regional pressure-volume curves to assess potentially recruitable lung volume and regional lung compliance [23,24]. Grant *et al*. used the regional filling characteristics to indicate tidal hyperinflation or recruitment [25]. However, these parameters summarizing the dynamic information obtained by EIT restrict to the time changes of regions of interest (ROIs), i.e. impedance pattern associated with different ROIs will not be described by these parameters.

Recently, the so-called global inhomogeneity (GI) index has been proposed and used to quantify tidal volume distribution within the lung [26]. The GI index represents an uncomplicated and efficient approach to summarize the complex pulmonary impedance distribution pattern by one number [16,27-29]. Up to now, the GI index has been evaluated for plausibility but an analysis of how it is affected by physiological factors is still missing. Since opening and closing of alveoli strongly influence ventilation distribution, we evaluated the influence of proportion of open lung regions measured by EIT on the GI index.

## Methods

### Patients and protocol

A total of 18 ARDS patients (12 male, 6 female; age 58 ± 14 years; height 177 ± 9 cm; weight 80 ± 11 kg (mean ± SD)) and 8 lung-healthy patients (5 male, 3 female; age 41 ± 12 years; height 177 ± 8 cm; weight 76 ± 8 kg) were analyzed retrospectively. Exclusion criteria were: age <18 years, pregnancy and lactation period, and any contraindication to the use of EIT (pacemaker, automatic implantable cardioverter defibrillator, and implantable pumps). The study was approved by the Ethics Committee of the Medical Faculty of the Christian Albrechts University in Kiel, Germany. Written informed consent was obtained from all patients or their legal representatives prior to the study.

The characteristics of the patients and the study protocol applied in the patients were previously described in detail [30]. The patients were sedated, paralyzed, and mechanically ventilated under pressure-controlled mode in supine position. Low-flow inflation was performed (Evita XL; Dräger, Lübeck, Germany) with a constant gas flow of 4 L/min starting at a zero end-expiratory pressure up to a tidal volume of 2 L or until a maximum airway pressure of 35 cmH_2_O was reached. Airway flow, pressure and volume were measured by the ventilator with a sampling rate of 126 Hz. EIT examinations were performed with the Goe-MF II EIT device (CareFusion, Höchberg, Germany). Sixteen ECG electrodes were attached on the chest circumference at the level of the fifth intercostal space. Alternating electrical currents (50 kHz, 5 mA peak-to-peak) were applied through adjacent pairs of electrodes in a sequential rotating process. The resulting potential differences were measured by the remaining electrode pairs. EIT raw data were acquired at 25 Hz and reconstructed offline with the Graz Consensus Reconstruction Algorithm for EIT, GREIT [31]. The reconstructed data were smoothed by a low-pass filter with 1 Hz cut-off frequency to suppress the influence of cardiac activity.

### Data analysis - recruitable lung regions

The lung regions in EIT images were defined during low-flow maneuver based on functional EIT scans (fEIT), which show the spatial distribution of the pixel linear regression coefficients [32]. Large positive values of linear regression coefficients indicate that the trends of the local (pixel) and total relative impedance curves are highly correlated. Regions with regression coefficients (R_pixel_) higher than 20% of the maximum value of all pixels (0.2 × max(R_image_)) in the corresponding fEIT images were considered to be lung regions. Thereafter, the maximum impedance amplitude during the low-flow inflation maneuver was determined in every pixel of the lung regions (max(Z_pixel, t1:tn_), where t1:tn was the time period of low-flow inflation). Lung region was defined as open (recruited) when, in the corresponding time course, the pixel amplitude exceeded 10% of the maximum amplitude, i.e. Z_pixel, tk_ > 0.1 × max(Z_pixel, t1:tn_) [30]. The remaining lung regions at that time point were recruitable. The percentage of recruitable lung regions was defined as 100% minus the number of recruited pixels divided by the total number of pixels in the lung regions.

### Data analysis - the GI index

The calculation of GI index has been described in previous studies [16,26]. In the present study, we modified the original GI index to adapt to the low-flow inflation. In brief, a differential image is first generated using EIT scans at two selected time points (not necessary to be end points of inspiration and expiration as defined in previous studies). Then the median impedance value within the predefined lung regions is calculated. The sum of the absolute difference between median value and every pixel value is considered to indicate the variation of ventilation distribution. Subsequently, it is normalized to the sum of the impedance values within the lung regions, in order to make the GI index universal and inter-patient comparable (Eq. 1).

(1) $$\mathrm{GI}=\frac{\underset{x,y\in \mathrm{lung}}{\sum }\left|DI_{\mathrm{xy}}-\mathrm{Median}\left(DI_{\mathrm{lung}}\right)\right|}{\underset{x,y\in \mathrm{lung}}{\sum }DI_{\mathrm{xy}}}$$

where *DI* is the value of the differential impedance in differential images; *DI*_*xy*_ denotes the *DI* of pixel *xy* in the identified lung regions; *DI*_*lung*_ are *DI* of all pixels in the lung regions under observation. In the present study, we modified the GI index slightly. Since the sampling rate of EIT scans was 25 Hz, the calculation of the GI index began one second after the onset of inflation. A series of GI indices was calculated during the inflation based on the differential images between time points t_26_ to t_1_, t_27_ to t_1_, t_28_ to t_1_, …, t_n_ to t_1_ (t_1_, the first EIT scan during inflation; t_n_, the *n*th EIT scan, n = 26, 27, …, until the end of inflation). The percentages of recruitable lung regions at corresponding time points were calculated and compared with GI index values using linear regression. To check for the effect of cardiac related changes in electrical impedance, the GI indices were calculated using both the low-pass filtered and unfiltered EIT data.

### Data analysis - respiratory system elastance

During low flow inflation, inspiratory volume was equally divided into 10 slices. Since the airway flow rate was constant during the maneuver, the respiratory system elastance (E_rs_) was calculated within each slice with the modified first order equation of motion:

(2) $$P_{\mathrm{aw},i}=E_{\mathrm{rs},i}\times V_{i}+k_{i}$$

where *P*_*aw,i*_ and *V*_*i*_ represent the airway pressure and volume within slice *i* (*i =* 1, 2, … 10). *k* is a constant, containing the resistive part of pressure drop. The volume level at the middle of each slice was identified and matched with the time point in the EIT measurements. Ten GI index values were determined at the corresponding time points.

### Statistical analysis

Data analysis was performed using MATLAB 7.2 (The MathWorks Inc., Natick, MA, USA). The Lilliefors test was used for normality testing. For normally distributed data, results were expressed as mean values ± standard deviations. Otherwise, data were presented as median and quartiles. The correlations between GI index and the percentages of recruitable lung regions, between GI index and E_rs_, between GI index and airway pressure, between GI index and lung volume were examined. Mediation analysis was performed to confirm that percentage of recruitable lung regions was a mediator variable for the observed relationship between GI index and airway pressure, between GI index and lung volume. Median GI values and median E_rs_ values of patients in ARDS group were compared to that in lung-healthy group with Mann–Whitney U test. A *P* value < 0.05 was considered to be significant.

## Results

**Figure 1:**
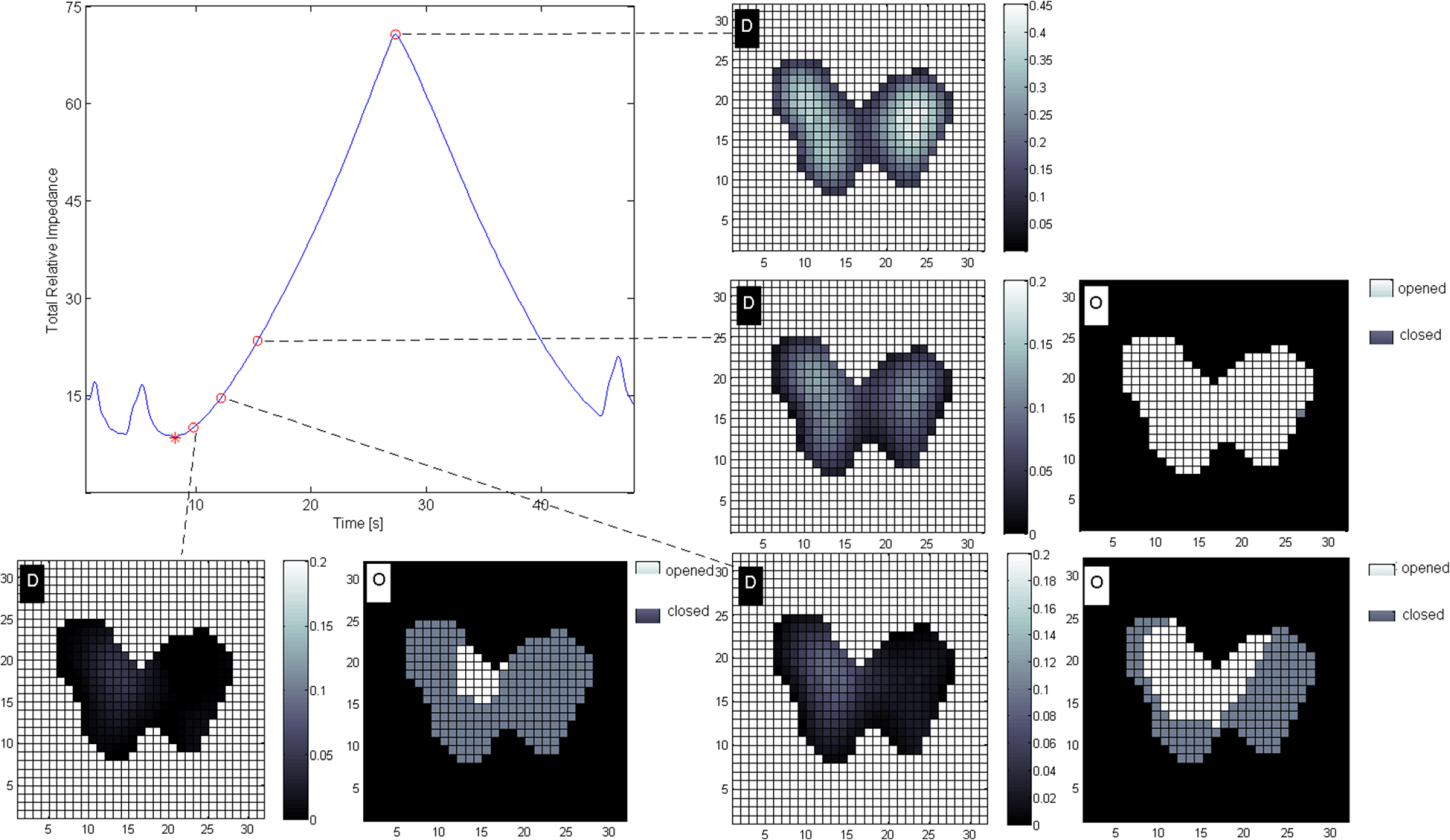
**Differential images (D) and the regional lung opening maps (O) during low-flow inflation.** Differential images show the impedance differences of lung region pixels between the EIT scans acquired at time points highlighted on the total relative impedance curve (left top) with red open circles and the EIT scan at the beginning of the inflation maneuver highlighted by a red asterisk. At the corresponding time points, open lung regions are shown in white in the regional lung opening maps. The highlighted time points from left to right are: the beginning of the low-flow inflation, 85%, 50%, 0% of the recruitable lung regions, and the end of inflation. Color bars indicate the magnitude of relative impedance values in each pixel.

**Figure 2:**
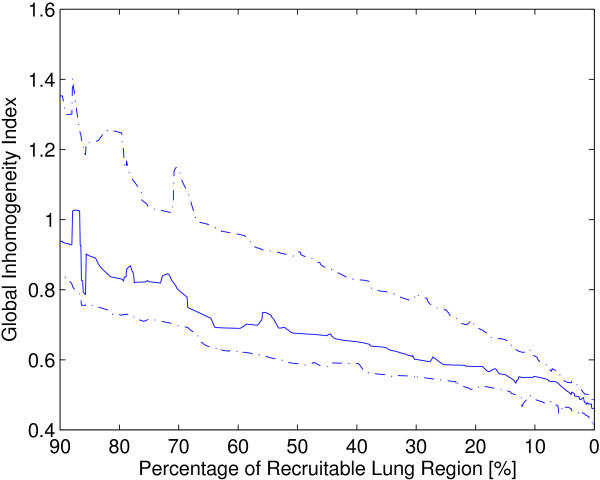
**Correlation of the GI indices and percentages of recruitable lung regions.** The aggregate trend of the GI indices and percentages of recruitable lung regions are summarized from all 18 ARDS patients during low-flow inflation. The line in the middle and the dashed lines above and below represent the median, upper and lower quartile respectively.

Figure 1 shows differential images and regional lung opening maps during low-flow inflation in one ARDS patient. The calculation of the GI index was based on these differential images. At low level of inflation, only a small proportion of all lung regions was recruited and, thus, ventilation distribution was inhomogeneous. While the number of open lung regions increased (percentages of recruitable lung regions decreased), ventilation distribution became more homogeneous, as indicated by the decrease of the GI index. Although the individual regression coefficients varied among different patients (the lower, median and upper quartile were 0.65, 0.74 and 1.04 respectively; Figure 2), the GI index and percentages of recruitable lung regions were highly correlated (*r*^*2*^*=* 0.88 ± 0.08, *p <* 0.01). Similar results were obtained for lung-healthy patients (*r*^*2*^*=* 0.92 ± 0.05, *p <* 0.01).

**Figure 3:**
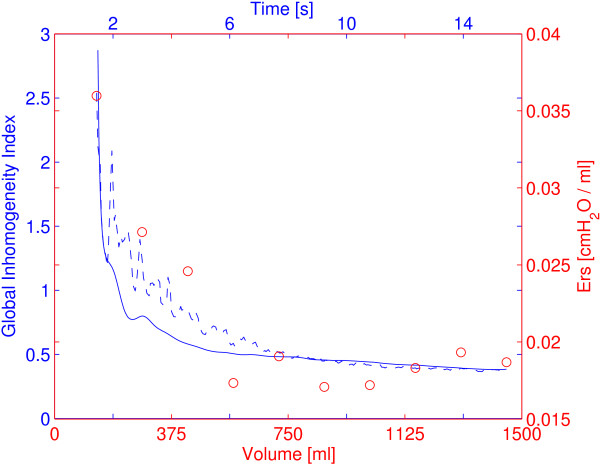
**Comparison of GI index values and respiratory system elastance.** The values of respiratory system elastance (E_rs_) are indicated with red circles. The GI index values with (blue solid line) and without (blue dashed line) low-pass filtering of cardiac related signals are plotted. Periodic cardiac activities can be recognized. The same patient as in Figure 1 is shown.

Figure 3 presents a comparison of the GI index values with and without low-pass filtering of cardiac related signals. The period of cardiac activity in this ARDS patient could be identified in the curve as 0.6 second. E_rs_ of the same patient at different ventilation levels were also shown in Figure 3. E_rs_ and sampled GI index values were highly correlated in 16 out of 18 ARDS patients (*r*^*2*^*=* 0.84 ± 0.13, *p <* 0.01). Comparable results were found in 6 out of 8 lung-healthy patients (*r*^*2*^*=* 0.84 ± 0.07, *p <* 0.01).

The GI index values were also significantly correlated with other parameters such as airway pressure and lung volume (*p <* 0.01). It was confirmed with mediation analysis that percentages of recruitable lung regions mediated the relation between airway pressure and GI index, between lung volume and GI index (regression coefficient decreased significantly, *p <* 0.01).

**Figure 4:**
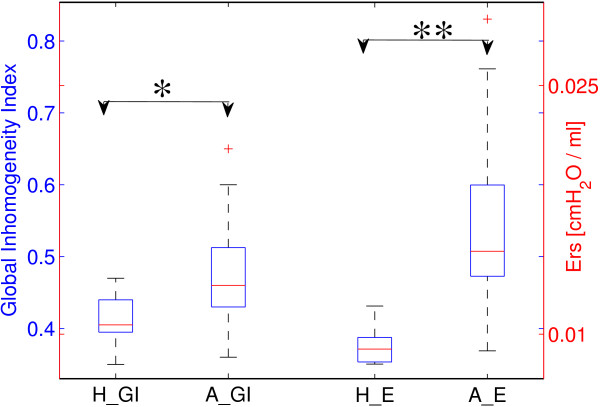
**Comparison of GI index values and respiratory system elastance in lung-healthy and ARDS groups.** H_G, A_G: median of GI index values in both lung-healthy (H) and ARDS (A) groups; H_E, A_E: median of respiratory system elastance (E_rs_) in both lung-healthy (H) and ARDS (A) groups. The boxes mark the quartiles while the whiskers extend from the box out to the most extreme data value within 1.5*the interquartile range of the sample. *, p < 0.05; **, p < 0.01.

Median GI values and median E_rs_ values were calculated for every patient in both ARDS and lung-healthy groups during low-flow maneuver (Figure 4). Significant differences were found in GI (0.41 ± 0.04 for lung-healthy and 0.52 ± 0.21 for ARDS, *p <* 0.05) and E_rs_ values (0.009 ± 0.001 cmH_2_O/ml for lung-healthy and 0.017 ± 0.008 cmH_2_O/ml for ARDS, *p <* 0.01) between the two study groups.

## Discussion

In the present study, we examined the effect of heart-beat, lung inflation and the degree of lung opening on GI index during a low-flow inflation maneuver and confirmed that the GI index was strongly influenced by the fraction of open lung areas in mechanically ventilated patients. We also confirmed that GI index values in lung-healthy and ARDS patients were significantly different.

The GI index has been proposed to monitor ventilation distribution [27,28], progression of obstructive lung disease [29], and to guide ventilator therapy [16]. Previously observed differences in the GI index between healthy volunteers and patients with various lung diseases [16,26,29] may be partly explained by the findings of the present study. In particular, lung regions of healthy volunteers open at low airway pressures (or even do not collapse at all) so that the GI index reaches a lower value during tidal ventilation. In patients, atelectatic lung regions may not be completely recruited during tidal ventilation so that the patients exhibit higher GI index values.

The GI index was originally calculated for tidal ventilation using differential EIT images between end-inspiration and end-expiration [26]. In a previous [29] and the present study, we found that a modified GI index can be used to assess the inhomogeneity at different lung volume levels. In the present study, the GI index was further modified to a quasi-continuous calculation. This enables us to closely monitor ventilation during prolonged inspiration and expiration. The same lung regions were used for GI calculation during whole low-flow inflation so that the values were intra-patient comparable. A limitation of the presented calculation compared to the original one is that regional overdistension cannot be captured in the serial differential images. Referencing to the baseline image, overdistended regions are considered to be “ventilated”. In the experimental setup, the low-flow inflation was terminated when a maximum airway pressure of 35 cm H_2_O was reached, to minimize the risk of VILI. E_rs_ values didn’t increase during low-flow maneuver (Figure 3), which indicated that the used threshold pressure was not sufficient to induce significant overdistension that was not identified by the lung mechanics measurement. Therefore, the influence of regional overdistension on the presenting calculation was minimum. Although a previous study [16] indicated that the GI index may be influenced by regional overdistension, their correlation should be systematically investigated with recruitment maneuvers and titration of positive end-expiratory pressure in further studies.

We selected ARDS patients since their lungs are heterogeneously affected and their alveoli tend to collapse at low end-expiratory airway pressures [33]. With a low-flow inflation maneuver, the relation between the GI index and the fraction of open lung regions (Figure 2), and between the GI index and E_rs_ (Figure 3) can be easily observed. The originate of GI index and open lung regions are different, although they are both derived from EIT images: the GI index depicts the relationship among pixels at certain time point; for open lung region calculation, impedance change of every single pixel in the lung region against time is traced.

We included also lung-healthy patients as control group. We found a similar trend to ARDS group that GI index decreased as ventilated regions increased. However, since recruitable lung regions occurred to a lesser degree in lung-healthy than in ARDS patients, both GI index and E_rs_ values were significantly lower in lung-healthy than in ARDS patients (Figure 4).

Continuous calculation of ventilation inhomogeneity is contaminated by cardiac-related signals in EIT data (Figure 3). This problem is solved by applying an adequate low-pass filter to the data to eliminate the signal component related to the heart beat, so that the resulting GI index is more plausible and comparable to E_rs_.

The correlations of the GI index and E_rs_ were not significant in two ARDS and two lung-healthy patients. The reason may be the limited number of E_rs_ estimates during the low-flow inflation in each patient, and sensitivity of the correlation to noise. However, because of the respiratory signal quality delivered by the ventilator, it was not possible to calculate more E_rs_ estimates.

One limitation of the study is that the gold-standard of identifying collapsed lung regions, namely CT scans, was missing. Due to radiation, CT is not a suitable bedside tool and indeed, there is no well established tool available for measuring recruitment/derecruitment dynamically. Besides CT scanning for study purposes would not be ethically justifiable. We used lung mechanics and the area of open lung measured in EIT to estimate the degree of recruitment. We confirmed that airway pressure increased the area of open lung, which influenced the GI index value. Further, we were not certain if all collapsed lung regions were recruited at a pressure level of 35 cm H_2_O [34]. These non-aerated regions would not be recognized as lung regions in fEIT and not be included into analysis. Identification of non-aerated lung regions in EIT measurements remains an important research topic and deserves further investigation. Nevertheless, patients with higher percentage of potential recruitable lung have higher mortality [34]. Thus, we focused on recruitable lung regions in the present study.

As discussed in our previous study [30], the identification of open lung regions with a threshold of 10% of the maximum local pixel amplitude of relative impedance change may slightly underestimate the true percentage of regional lung opening. This may influence the values of regression coefficients but not the conclusion that the GI index highly correlates with regional lung recruitment. Since it is not a well accepted method to estimate the recruitable regions, we also compared the GI index with respiratory system elastance and obtained a similar conclusion. The values of regression coefficients varied in different patients (Figure 2) indicating that individual therapeutic strategies should be developed to optimize the mechanical ventilation treatment. In practice, given the character of the GI curves presented in Figure 2, a simplified low-flow maneuver with half of duration or scanning at 3–4 different PEEP levels is sufficient to depict the GI index-recruitable regions curves, which may be helpful for development of individual therapeutic strategies.

## Conclusions

We conclude that the GI index is a reliable measure of ventilation heterogeneity highly correlated with lung recruitability measured with EIT. The GI index may prove to be a useful EIT-based index to guide ventilation therapy.

## Competing interests

The authors declare that they have no competing interests.

## Authors’ contributions

ZZ designed the study, analyzed the data and drafted the manuscript. SP carried out the data measurement. IF contributed to study design and revised the manuscript critically. UML gave valuable advices and contributed to writing. KM contributed to study design, data analysis and writing. All authors read and approved the final manuscript.
